# Aging and Neurodegeneration: A Tangle of Models and Mechanisms

**DOI:** 10.14336/AD.2016.0312

**Published:** 2016-03-15

**Authors:** Sasanka Chakrabarti, Kochupurackal P. Mohanakumar

**Affiliations:** 1 ICARE Institute of Medical Sciences and Research, Haldia, West Bengal, India; 2 Inter University Centre for Biomedical Research & Super Speciality Hospital, Mahatma Gandhi University Campus at Thalappady, Kottayam, Kerala, India

**Keywords:** aging, oxidative stress, mitochondrial dysfunction, Alzheimer's disease, autophagy, amyloid beta peptide

## Abstract

The research on aging and age-related diseases, especially the neurodegenerative diseases, is on the fast track. However, the results have so far not been translated to actual benefit for the patients in terms of treatment or diagnosis of age-related degenerative diseases including those of the CNS. As far as the prevention of the cognitive decline during non-pathological aging is concerned, there is nothing much to offer other than calorie restriction and physical exercise. Needless to say, the benefits are not up to our expectations. However, over the years at the experimental level it has been possible to identify several cellular and molecular mechanisms that are intricately associated with aging in general and neurodegenerative diseases in particular. These include oxidative stress and altered redox-signaling, mitochondrial dysfunction, inflammation, proteotoxicity and altered gene expressions. These inter-dependent pathways mediate cellular senescence and often culminate in programmed cell death like apoptosis and autophagy, and in the context of brain these changes are manifested clinically as cognitive decline and pathologically as neurodegeneration. This special issue provides the readers with glimpses of this complex scenario from different angles primarily in the context of brain and also attempts to identify the potential drug targets against neurodegenerative diseases.

The eight articles in this special issue appear a bit disparate at the first sight, but the common theme is easily discernible on scrutiny. All the articles are related to causes, consequences, models and mechanisms of aging and age-related diseases in general and neurodegeneration and neuroprotection in particular. The importance of the topic cannot be too emphasized in a world where the percentage of aged population is on the rise continuously causing a huge burden on every nation in terms of finances and health care support. For majority of the articles in this series, the main context is the brain aging or Alzheimer's disease or other kinds of brain damage, but a few articles have addressed the process of aging and age-related diseases on a larger canvas. Several of these reviews were presented and discussed in an international conference (NEUROCON 2015) held last year at Haldia, India [1]. These reviews have not attempted to present a comprehensive and cohesive view of our understanding of aging and neurodegeneration, but instead have highlighted several novel facets of these pathophysiological processes which may lead us to new avenues of protective therapy especially against neurodegeneration. The authors do not posit any radical departure from our existing dogma of neurodegenerative process, but explore new areas within the established conceptual framework.

The manifest common end point of brain aging and AD is the impairment of memory and cognition, and at the molecular and cellular level the similarity is very striking between these two conditions. For example, mitochondrial dysfunction, oxidative stress, inflammatory response and proteotoxicity are common themes in AD and brain aging [2,3,4,5]. However, there are important differences in the severity of these alterations in non-pathological brain aging and AD. What is not known why the aging brain progresses to AD with extensive neurodegeneration in some cases but not in others. The triggers that lead to sporadic AD in the absence of known mutations of familial AD are only tentative, and there are no clear molecular links between epidemiological and experimental data [6]. The identifications of familial AD mutations in *APP, PS 1* and *PS2* genes have given a big boost to the hypothesis of proteotoxic mechanism mediated by the oligomers of amyloid beta peptide, often called the Amyloid Cascade Hypothesis, in explaining the neurodegeneration of the sporadic AD. Thus, the experimental AD research has produced a surfeit of mechanistic pathways triggered by amyloid beta peptide oligomers that culminate in the apoptotic or autophagic death of neurons [7, 8]. Despite this the therapeutic approaches targeting APP and amyloid beta peptide in AD have consistently failed. Thus alternative approaches in understanding the neurodegenerative process from the known mechanisms of cellular senescence may eventually lead to newer therapeutic possibilities in tackling the brain deficits of aging and AD.

Mitochondrial dysfunction and oxidative stress as mentioned already are central mechanisms involved in brain aging and AD pathogenesis, and two articles in this issue deal with these aspects. As far as the oxidative stress is concerned, Sesti has emphasized in his review that instead of studying the generalized oxidative damage markers, the identification of specific oxidative alterations in ion channels in the brain would be more revealing [9]. Thus, reactive oxygen species (ROS)-mediated modifications of delayed-rectifier and Ca^2+^ activated K^+^ channels could be linked to altered electrophysiology of neurons with clear implications in brain aging and AD [9]. Onyango et al. have analyzed in depth the bioenergetics and biogenesis of mitochondria and suggested how calorie restriction, exercise or drugs like metformin and resveratrol would be beneficial in maintaining mitochondrial homeostasis [10]. This has obvious implications in the context of brain aging and AD. The concept of mitohormesis as an adaptive measure could be important in planning antioxidant-based therapy for brain aging or AD [10]. In this context, the article by Redmann et al. has added significance, and the authors have reviewed in details the conflicting ideas and evidence regarding the role of oxidative stress, ROS signaling, mitochondrial functions and autophagy in the process of aging and neurodegeneration [11]. The authors suggest that the failure of antioxidant therapy in neurodegenerative diseases may be related to the interference with the physiological redox signaling pathways, and they further propose that the process of autophagy may be the key regulator of cellular bioenergetic efficiency and survival during oxidative and proteotoxic stress [11]. This automatically implies that stimulation of autophagic process may provide neuroprotection, but the scenario could be murky if uncontrolled autophagy leads to elimination of essential proteins and functional organelles [11]. Nevertheless, trehalose which stimulates autophagy provides protection in several models of neurodegeneration [11]. Another novel method of neuroprotection has been suggested in the context of traumatic brain injury and the actions of APP and its derivative sAPPα [12]. In this article, Plummer et al. have reviewed the neuroprotective function of APP in various model systems and its role in cell-cell interactions, cell adhesion and synaptic modification which all could be important in the protective functions of APP in traumatic brain injury [12]. It is interesting that the heparan binding domain within the growth-factor like domain of APP is responsible for the neuroprotective action of the latter in traumatic brain injury [12]. It is tempting to speculate that the increased production of APP and amyloid beta peptide in AD is a protective response of the brain against the backdrop of neurodegeneration. Two genetic defects of compromised α-oxidation of fatty acids (Refsum disease) and impaired oxidation of very long chain fatty acids (Adrenoleukodystrophy) are characterized by profound neurodegeneration. These two disease states have been reviewed in-depth by Schönfeld and Reiser from the point of view of altered energy metabolism and ROS production as well as mitochondrial structural and functional derangement, and the links to neurodegeneration have been indicated [13]. The authors suggest that the accumulation of very long chain fatty acids occurs in AD brain contributing to neurodegeneration, and this is a refreshingly new concept. From a somewhat different angle, another interesting review paper has looked in to various alterations in gene expressions and epigenetic changes that could be related to cognitive impairment of brain aging and AD [14]. The authors suggest that alterations in DNA methylases and histone deacetylases may be the key regulators of global gene expression changes in brain aging and AD and therefore these should be the ideal targets for drug development [14].

Hypoxia induced changes in gene transcription through activation of the transcription factor HIF-1α and subsequent activation of a set of histone lysine demethylases causing epigenetic changes in chromatin have been analyzed in great details in the review article by Salminen et al. [15]. While acute hypoxic stress activates HIF-1α leading to enhanced transcription of many genes involved in cell survival, energy metabolism, mitochondrial functions and autophagy, the chronic hypoxic stimulus may aggravate cellular aging process and age-related tissue damage, and this latter phenomenon may be mediated by the activation of histone lysine demethylases contributing to epigenetic changes epigenetic changes [15]. Given the fact that chronic hypoxia from cerebrovascular ischemic changes can contribute to AD pathology and clinical features in different ways, the role of HIF-1α and histone lysine demethylases needs to be explored thoroughly in this disease condition [16]. A prospective epidemiological study in this issue shows that the presence of complex aortic plaque increases the risk for developing recurrent ischemic cerebrovascular events [17]. The importance of this finding may be evaluated further in AD subjects especially in the mixed type with prominent cerebrovascular changes.
